# Supplementary material for: Mechanisms of triplet energy transfer across the inorganic nanocrystal/organic molecule interface
Source: Nat Commun. 2020 Jan 7;11:28. doi: 10.1038/s41467-019-13951-3 (PMC6946700; doi:10.1038/s41467-019-13951-3)
Supplement: Supplementary file 1 — Supplementary Information [file 41467_2019_13951_MOESM1_ESM.pdf]

Supplementary Information

Mechanisms of triplet energy transfer across the inorganic nanocrystal/organic molecule interface

Luo et al.

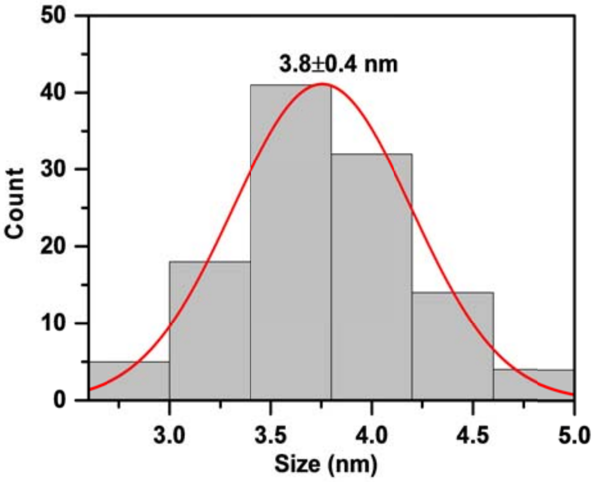

Supplementary Figure 1. Size distribution of $CsPbBr_3$ NCs.

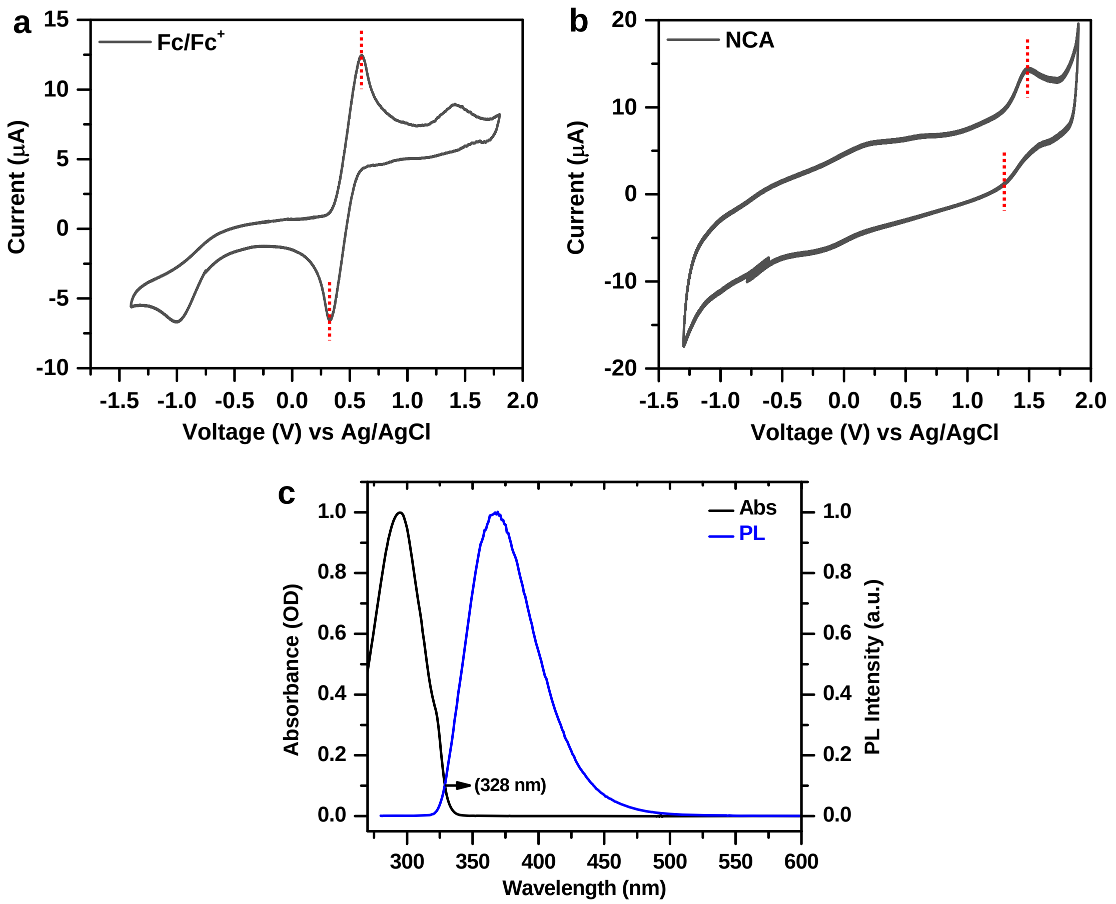

Supplementary Figure 2. Energy levels of NCA. CV curves of Fc/Fc^+ standard (a) and NCA (b) measured at RT. The CVs were performed in acetonitrile at a scan speed of 100 mV s^{-1}. The redox couples labeled in (b) can be assigned to the HOMO or oxidation potential of NCA. The value of the oxidation potential energy vs. vacuum can be calculated as: E_{ox} = -[V_{ox}-V(Fc/Fc^+)+4.8]eV=-[(1.5+1.3)/2-(0.3+0.6)/2+4.8]=-5.8 eV. In this equation, the redox potentials of the molecules were calculated as the mean values of the cathodic and anodic peaks. 4.8 is the potential of Fc/Fc^+ vs. vacuum. Because the LUMO or reduction potential of NCA was not obtained in the used electrochemical window. We calculate it using: $E_{red} = E_{ox} + E_g$, with E_g being the optical gap of the molecule. E_g of NCA can be determined from the the crossing point between the normalized absorption and luminescence spectra in (c). E_{red} of TCA can thus be calculated as: $E_{red} = E_{ox} + E_g$ = -5.8 + 3.8 = -2.0 eV.

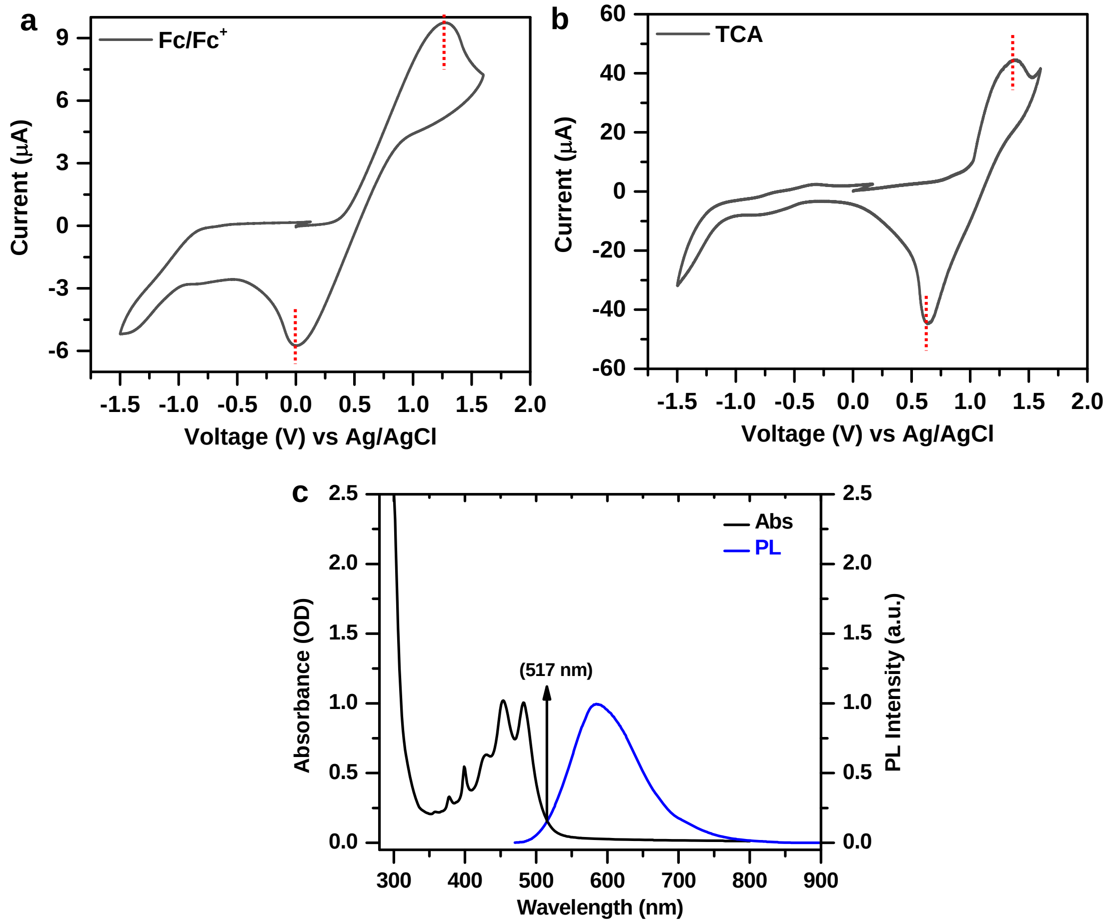

Supplementary Figure 3. Energy levels of TCA. CV curves of Fc/Fc^+ standard (a) and TCA (b) measured at RT. The CVs were performed in dichloromethane at a scan speed of 100 mV s^{-1}. The redox couples labeled in (b) can be assigned to the HOMO or oxidation potential of TCA. (c) Normalized absorption and luminescence spectra of TCA used to determine its E_g. E_{ox} of TCA can be calculated as: E_{ox} = $-[V_{ox}-V(Fc/Fc^+)+4.8]$eV=-[(1.37+0.63)/2-(1.29+0.01)/2+4.8]=-5.1 eV. E_{red} of TCA can be calculated as: $E_{red} = E_{ox} + E_g = -5.1 + 2.4 = -2.7$ eV.

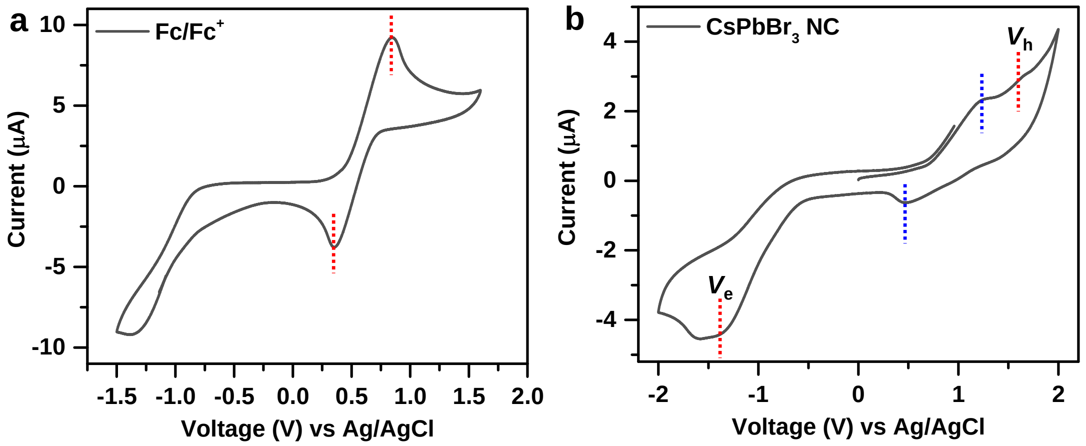

Supplementary Figure 4. Energy levels of NCs. CV curves of Fc/Fc^+ standard (a) and $CsPbBr_3$ NCs (b) measured at RT. The CVs were performed in acetonitrile/toluene mixture (1:4 v/v) at a scan speed of 100 mV s^{-1}. V_e and V_h are labeled in (b) and the extra peaks labeled by blue dashed lines were likely due to additional species in the solution such as the unreacted Pb-oleate.[1] Also note that E_e and E_h are irreversible peaks so that their values are determined from single peaks instead of mean values of cathodic and anoic peaks, which are consistent with previous CV studies on NCs.[1-3] As such, $E_e = -[V_e - V(Fc/Fc^+) + 4.8]$eV = -[-1.4-(0.4+0.8)/2+4.8] = -2.8 eV; $E_h = -[V_h - V(Fc/Fc^+) + 4.8]$eV = -[1.5-(0.4+0.8)/2+4.8] = -5.7 eV. The difference between E_e and E_h is 2.9 eV, which is higher than the optical gap of the NCs (~2.7 eV) because of a strong electron-hole coulomb binding energy in NCs.

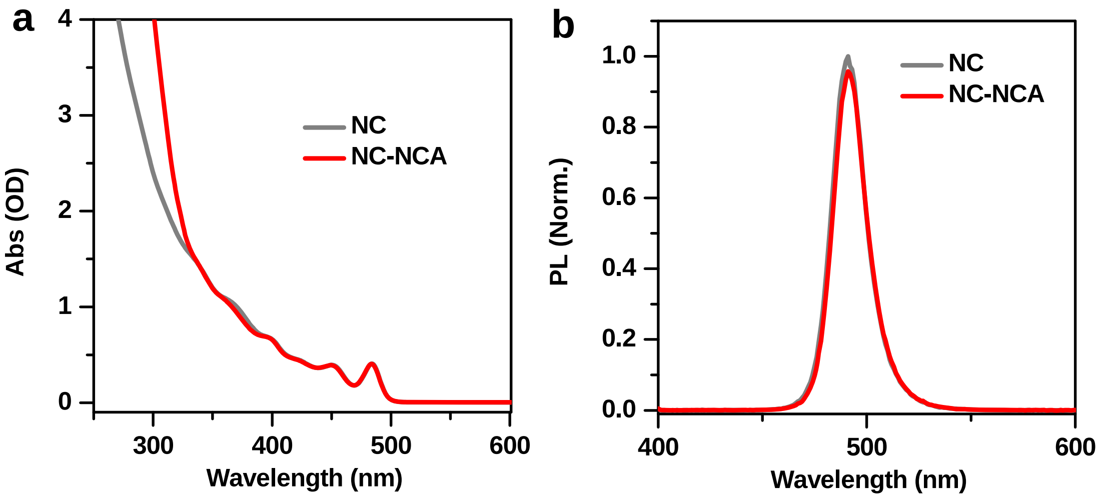

Supplementary Figure 5. Large-size NC-NCA. (a) Absorption and (b) PL spectra of larger-size $CsPbBr_3$ NCs (gray) and NC-NCA complexes (red). PL was acquired using 400 nm excitation.

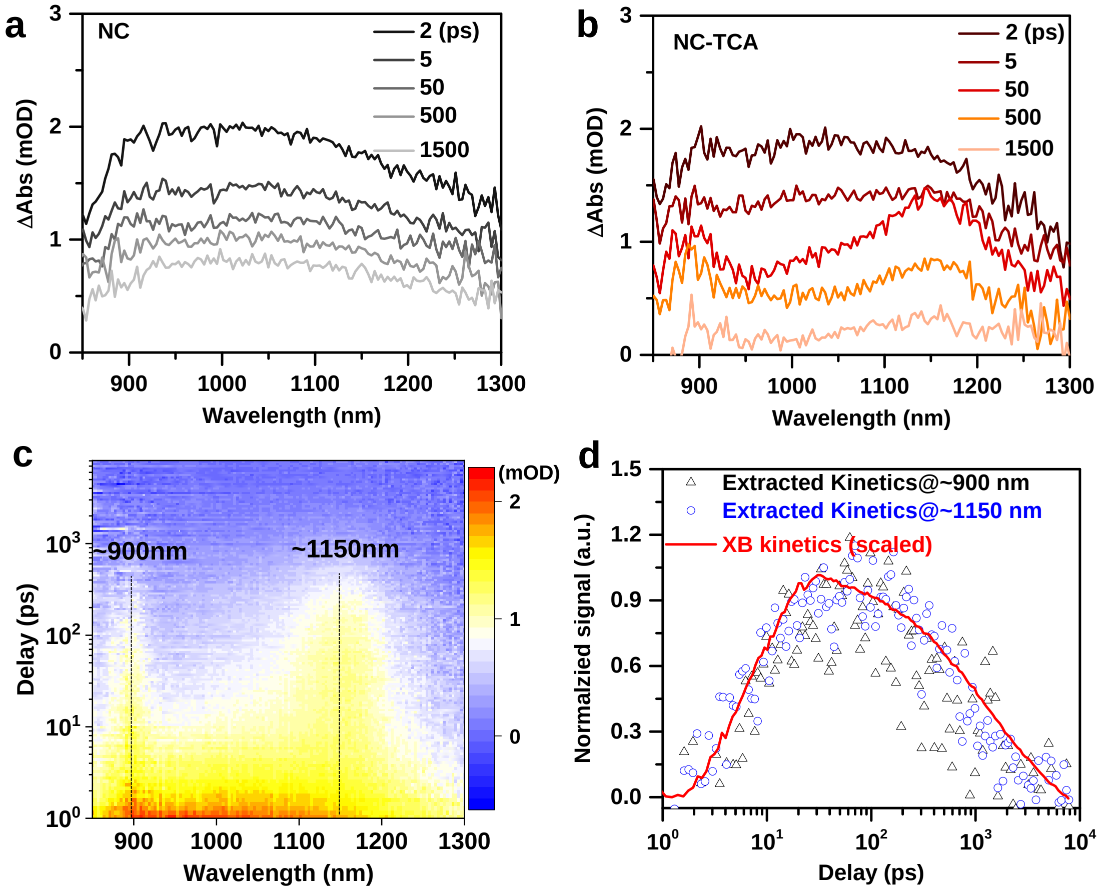

Supplementary Figure 6. NIR TA spectra and kinetics. (a,b) NIR TA spectra of $CsPbBr_3$ NCs (a) and NC-TCA complexes (b) at indicated time delays following the excitation by a 340 nm pulse. (c) 2D pseudo-color plot of the NIR TA spectra of NC-TCA complexes. (d) Comparison of the extracted radical kinetics at ~900 (black triangles) and ~1150 nm (blue circles) of TCA and the scaled XB kinetics of NCs (red solid line).

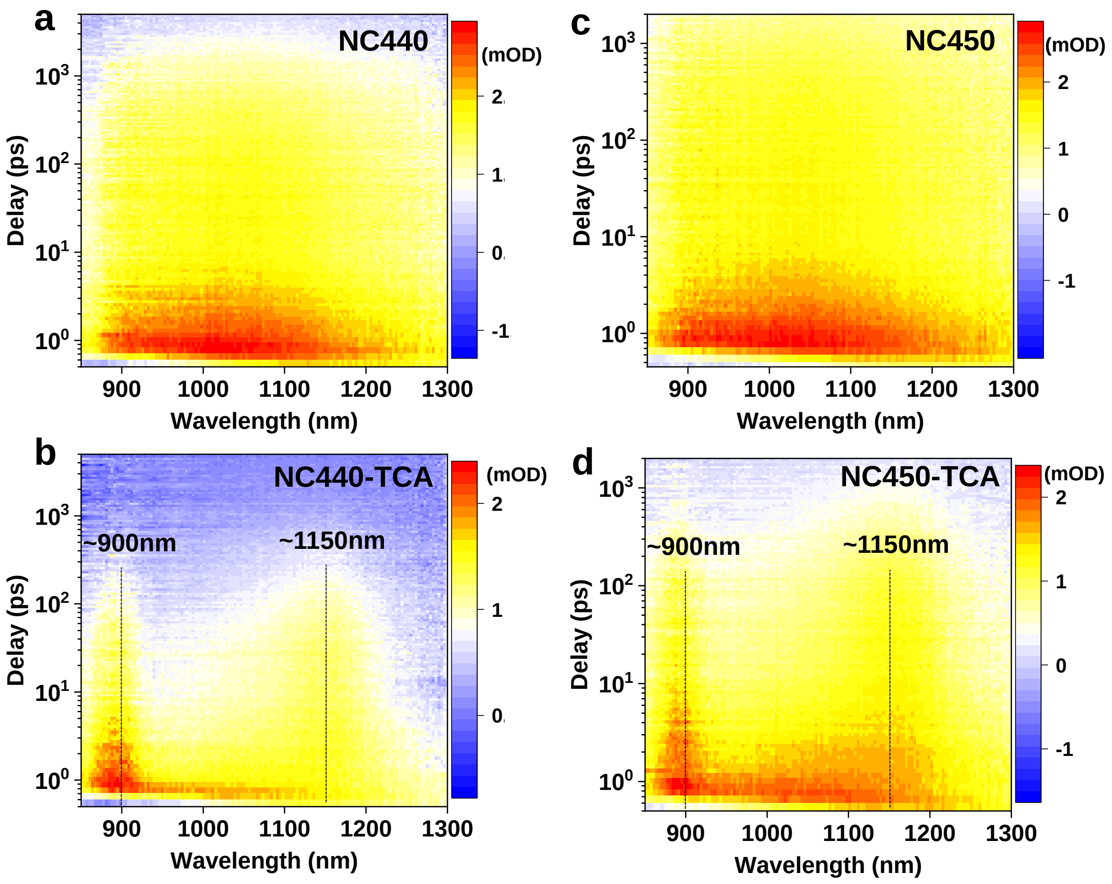

Supplementary Figure 7. Different-size NC-TCA NIR data. 2D pseudo-color plots of NIR TA spectra of $CsPbBr_3$ NCs (a) and NC-TCA complexes (b) with the lowest energy absorption peak at 440 nm. (c,d) The same plots as (a) and (b) but for NCs with the lowest energy absorption peak at 450 nm.

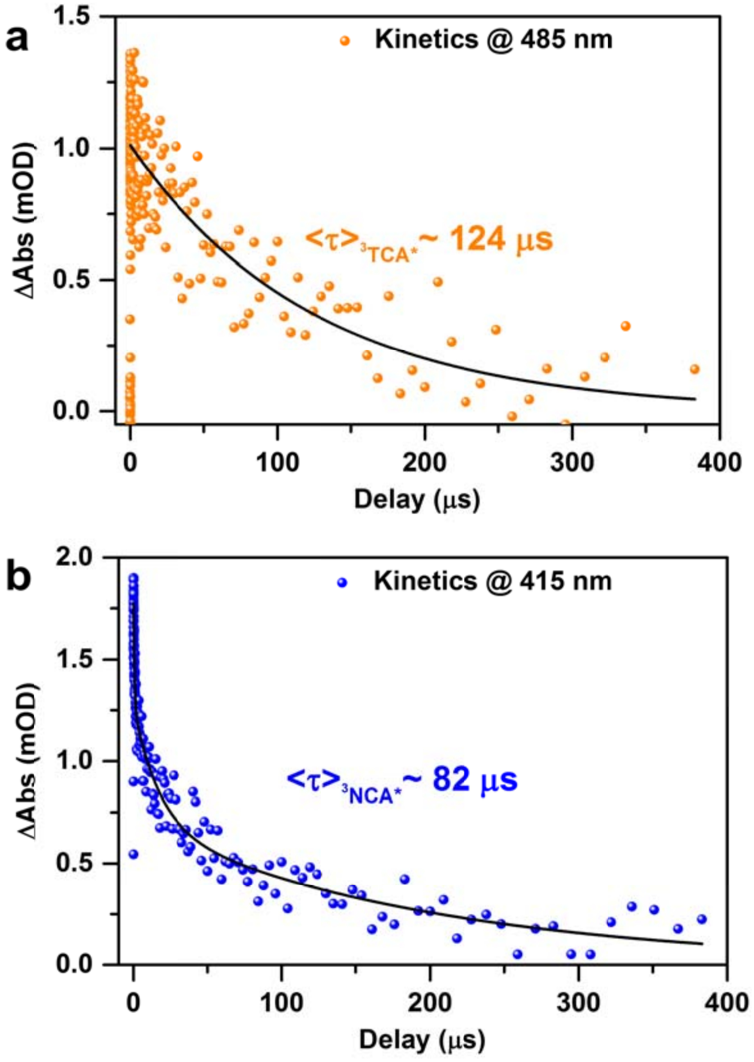

Supplementary Figure 8. Triplet lifetime. (a) TA kinetics monitored for the photoinduced absorption of ^{3}TCA* at 485 nm and a fit to the kinetics with a lifetime of ~124 μs. (b) TA kinetics monitored for the photoinduced absorption of ^{3}NCA* at 415 nm and a fit to the kinetics with a lifetime of ~82 μs.

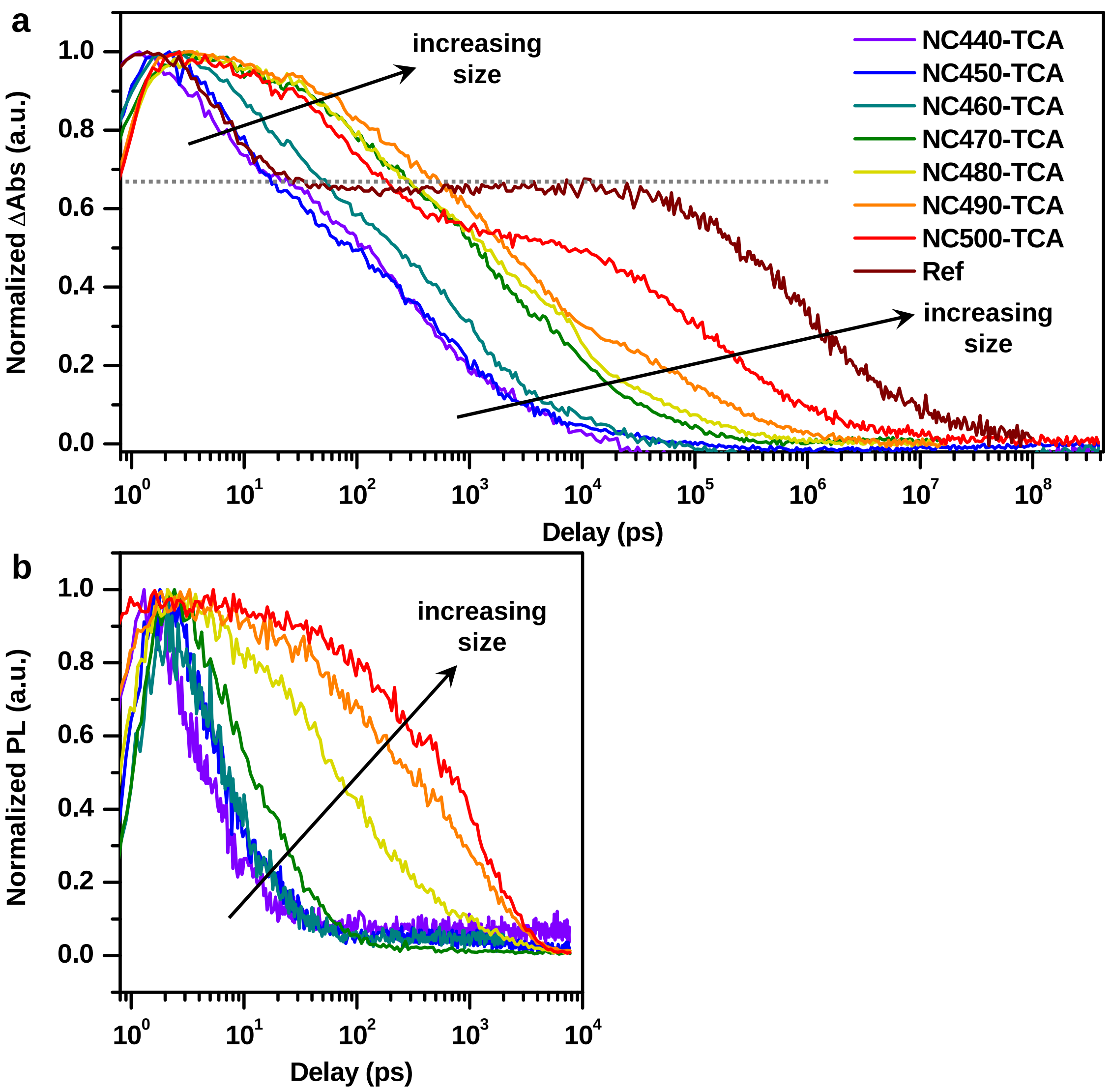

Supplementary Figure 9. Size-dependent NC-TCA kinetics. Normalized XB (a) and TR-PL (b) kinetics of a series of NC-TCA complexes with different NC sizes. The sizes of these NCs are tuned from ~2.6 to 8.5 nm, corresponding to first-exciton absorption peaks from 440 to 500 nm which are used to label the NCs in the figure. The wine-colored line in (a) is from ref[4] which used a sample of ~10-nm $CsPbCl_xBr_{3-x}$ NCs. The TA data above and below the gray dashed line in (a) mostly reflect hole and electron transfer, respectively, whereas the TR-PL data in (b) measure hole transfer only. Both hole and electron transfer slow down with increasing NC sizes.

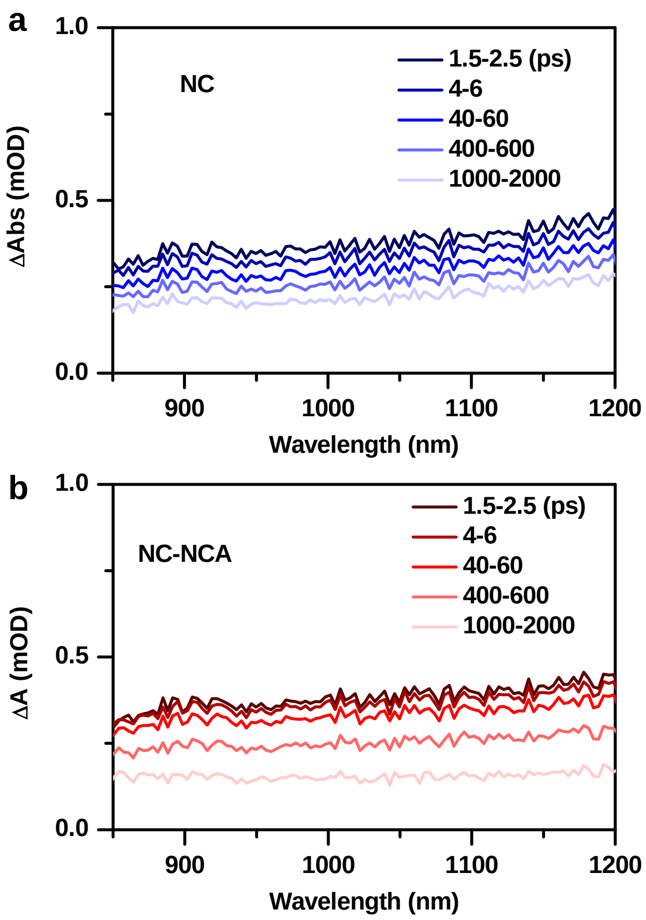

Supplementary Figure 10. NC-NCA NIR TA. NIR TA spectra of $CsPbBr_3$ NCs (a) and NC-NCA complexes (b) at indicated time delays following the excitation by a 400 nm pulse.

Supplementary Table 1. Free energy changes for charge transfer reactions.

	ΔG_{HT} (eV)[a]	ΔG_{ET1} (eV)[b]	ΔG_{ET2} (eV)[c]
NC-TCA	-0.3	0.4	-1.3
NC-NCA	0.4	1.1	-0.7

[a] Hole transfer from photoexcited NCs to ground state PAH; [b] Electron transfer from photoexcited NCs to ground state PAH; [c] Electron transfer from NC^- to PAH^+ to form a triplet. Note that because CV measurements for energy levels are in general accurate to 0.1 V,[5] we report numbers to one decimal place.

Supplementary Table 2. Fitting parameters for TA and PL kinetics of pristine NCs

pristine NCs	τ_T (ps)<br>A_1 (%)	τ_X (ps)<br>A_2 (%)	τ_{CR} (ps)<br>A_1 (%)
	160±10<br>(22.0±1.4)	5000±146<br>(78.0±2.3)	16047±326<br>(22.0±1.4)

Supplementary Table 3. Fitting parameters for TA and PL kinetics of NC-TCA complexes

	hole transfer or	electron injection or triplet formation	Φ_{TET} (%)

	PL quenching				
NC-TCA	τ_{HT} (ps)<br>A (%)	$\tau_{ET,1}$ (ps)<br>A_1 (%)	$\tau_{ET,2}$ (ps)<br>A_2 (%)	$\tau_{ET,ave}$ (ps)	98.6
	8.9±0.8<br>(100.0±1.0)	452±21<br>(34.0±1.5)	2612±102<br>(66.0±1.2)	1878±74	

Supplementary Table 4. Fitting parameters for TA and PL kinetics of NC-NCA complexes

NC-NCA	$\tau_{PL,1}$ (ps)<br>A_1 (%)	$\tau_{PL,2}$ (ps)<br>A_2 (%)	$\tau_{PL,ave}$ (ps)	τ_{TET} (ps)	Φ_{TET} (%)
	155±4<br>(29.6±0.4)	1900±49<br>(70.4±1.0)	1383±36	2134±51	65.0

Supplementary Note 1. Calculation of charge transfer driving forces (-Δ*G*)

The charge transfer (CT) driving force is calculated from the free energy change between the reactant and product states. We follow the formalism in ref.[6] to calculate this free energy change.

Hole transfer from photoexcited NCs to ground state PAH. In the case of hole transfer from photoexcited NCs (NC*) to ground state polycyclic aromatic hydrocarbons (PAHs), *i.e.*, electron transfer from the PAH to the NC*, the reactant is NC*-PAH and its free energy is:

$$G_R = E_{\mathrm{NC*}} + E_{PAH} = E_{NC} + E_e - E_h + E_{e-h} + E_{PAH} \qquad (1),$$

where E_{NC} is the energy of the ground state NC, E_e and E_h are the energies of the first quantum-confined electron and hole levels, respectively, in the NCs, E_{e-h} is the electron-hole Coulomb binding energy (a negative value), and E_{PAH} is the energy of the ground state PAH molecule. The product state after hole transfer is $\mathrm{NC^{-}}$-$\mathrm{PAH^{+}}$ whose free energy is:

$$G_P = E_{NC} + E_e + E_{c(\mathrm{e})} + E_{PAH^+} + E_{c(\mathrm{h})} + E_{CS} \qquad (2),$$

where $E_{PAH}{}^{+}$ is the energy of the oxidized PAH, $E_{c(e)}$ is the charging energy of putting an electron in the NC, $E_{c(h)}$ is the charging energy of putting a hole in the PAH, and E_{CS} is the electron-hole binding energy in charge separated state $\mathrm{NC^{-}}$-$\mathrm{PAH^{+}}$ (a negative value). Therefore, the total free energy change of the hole transfer reaction is:

$$\begin{aligned}\Delta G_{HT} &= G_P - G_R = E_h - E_{e-h} + E_{c(e)} + E_{CS} - (E_{PAH} - E_{PAH^+} - E_{c(h)}) \\ &= E_h - E_{e-h} + E_{c(e)} + E_{CS} - E_{PAH/PAH^+}\end{aligned} \qquad (3),$$

where the term $E_{PAH} - E_{PAH^+} - E_{c(h)}$ is, by definition, the oxidation potential energy of the ground state PAH (E_{PAH/PAH^+} or E_{OX} shown in Fig. 1c).

Electron transfer from photoexcited NCs to ground state PAH. In this case, the reactant state is the same as Supplementary Equation 1 and the product state after electron transfer is NC^+-PAH^- whose free energy is:

$$G_P = E_{NC} + E_h + E_{c(\mathrm{h})} + E_{PAH^-} + E_{c(\mathrm{e})} + E_{CS} \qquad (4),$$

where E_{PAH^-} is the energy of the reduced PAH. The total free energy change of the electron transfer reaction is:

$$\begin{aligned}\Delta G_{ET1} &= G_P - G_R = -E_e - E_{e-h} + E_{c(\mathrm{h})} + E_{CS} + (E_{PAH^-} + E_{c(\mathrm{e})} - E_{PAH}) \\ &= -E_e - E_{e-h} + E_{c(\mathrm{h})} + E_{CS} + E_{PAH/PAH^-}\end{aligned} \qquad (5),$$

where the term $E_{PAH^-} + E_{c(e)} - E_{PAH}$ is, by definition, the reduction potential energy of the ground state PAH (E_{PAH/PAH^-} or E_{red} shown in Fig. 1c).

Electron transfer from NC^- to PAH^+ to form a triplet. In this case, the reactant state is the same as Supplementary Equation 2 and the product state after electron transfer is NC-^{3}PAH* whose free energy is:

$$G_P = E_{NC} + E_{^3PAH*} = E_{NC} + E_{PAH} + E_T \qquad (6),$$

where E_T is the energy difference between the PAH triplet and ground state and is triplet energy shown in Fig. 1c. The total free energy change of the reaction is:

$$\Delta G_{ET2} = G_P - G_R = -E_e - E_{c(\mathrm{e})} - E_{CS} + (E_{PAH} - E_{PAH^+} - E_{c(\mathrm{h})} + E_T) \\ = -E_e - E_{c(\mathrm{e})} - E_{CS} + E_{^3PAH^*/PAH^+} \quad (7),$$

where the term $E_{PAH} - E_{PAH^+} - E_{c(\mathrm{h})} + E_T$ is, by the Rehm-Weller approximation,[7] the reduction potential energy of the triplet excited state of the PAH ($E_{^3PAH^*/PAH^+}$ or $E_{red,T}$ shown in Fig. 1c). The Rehm-Weller approximation has been extensively used to estimate the redox potential energy of the singlet and triplet excited states using that of the ground state.[8-10]

Calculating energy terms. The electron-hole binding energy term ($E_{e\text{-}h}$) in the NCs was estimated using the energy levels determined from CV (E_e = -2.8 eV and E_h = -5.7 eV vs vacuum) and the exciton energy determined from the optical gap (E_X ~2.7 eV):

$$E_{e-h} = E_X - (E_e - E_h) = -0.2\,\mathrm{eV} \quad (8).$$

The NC charging energy $E_{c(e\ or\ h)}$ has been derived as:[11]

$$E_c(R) = \frac{0.786e^2}{8\pi\varepsilon_0\varepsilon R} + \frac{e^2}{8\pi\varepsilon_0\varepsilon_s R} \quad (9),$$

where ε is the effective dielectric constant of bulk $CsPbBr_3$ (~5 relative to the vacuum value of ε_0)[12], ε_{sol} is the effective dielectric constant of the solvent (1.9 for hexane). In its simplest form without accounting for the dielectric contrast effect, the electron-hole binding energy in the charge separated state (E_{CS}), assuming a point charge sitting on the surface of NCs, can be estimated as:

$$E_{CS}(R) = -\int_0^R dr \int_0^\pi d\theta \int_0^{2\pi} d\varphi \frac{r^2 \sin\theta e^2 \rho(r)}{4\pi\varepsilon\sqrt{r^2\sin^2\theta + (R - r\cos\theta)^2}} \quad (10),$$

where $\rho(r)$ is the charge density inside the NC and can be expressed as the following by assuming charge wavefunction for particle in a sphere of infinite depth:

$$\rho(r) = e\left[\frac{\sin(\pi r/R)}{r\sqrt{2\pi R}}\right]^2 \qquad (11).$$

The estimated $E_{c(e)}$, and E_{CS} for the 3.8-nm-diameter $CsPbBr_3$ NCs are ~0.26 and -0.13 eV, respectively.

With these energy terms calculated, we can calculate the free energy changes for the reactions described in Supplementary Equations 3, 5 and 7. The results are tabulated in Supplementary Table 1.

Supplementary Note 2. Kinetics fitting models

Model for pristine NCs. On the basis of the transient absorption (TA) and PL measurements, a kinetic fitting model is proposed as follows: upon photoexcitation of $CsPbBr_3$ NC, the exited-states (NC*) from a sub-ensemble of NCs quickly decay in 200 ps through ultrafast hole trapping to form charge separated states (NC^--$trap^+$) with a rate of k_T; the rest NC* decay via band edge exciton recombination to form ground state NC with a rate of k_X; the NC^--$trap^+$ slowly decay through charge recombination to form ground state NC with a rate of k_{CR}. Note that the TA and PL probe the contribution-weighted sum and the product of the electron and the hole, respectively. Electron and hole contributions to the XB signal in $CsPbBr_3$ NCs are ~75% and 25%, respectively. Thus, we fit the XB and PL kinetics in $CsPbBr_3$ NCs with the following multi-exponential decay functions:

$$S(t)_{NC,TA} = 0.25\cdot(A_1\cdot e^{-k_T t} + A_2\cdot e^{-k_X t}) + 0.75\cdot(A_1\cdot e^{-k_{CR} t} + A_2\cdot e^{-k_X t}) \quad (12),$$

$$S(t)_{NC,PL} = A_1\cdot e^{-k_T t} + A_2\cdot e^{-k_X t} \quad (13),$$

where A_1 and A_2 are the relative amplitudes. The fitting parameters are listed in Supplementary Table 2. The kinetic traces for TA and PL in Fig. 4b are simultaneously fitted using Supplementary Equations 12-13 using the same set of fitting parameters; note that the rate constants in the above equations are converted to time constants in the table.

Model for NC-TCA complexes. According to the TA and PL measurements, a kinetic fitting model is proposed as follows: upon selective photoexcitation $CsPbBr_3$ NCs in NC-TCA complexes, almost all band-edge holes transfer from NC to TCA within 50 ps to form charge separated states (NC^--TCA^+) with a rate of k_{HT}, which outcompetes both electron-hole recombination (5 ns) and hole trapping (160 ps). From 50 ps to ns timescale, the band-edge electrons in NC^- are injected into the triplet states of TCA^+ to form NC-$^3TCA^*$ with a rate of k_{ET}, while the TA signal of $^3TCA^*$ grows in. Thus, the hole transfer and PL quenching kinetics are simultaneously fitted using a mono-exponential decay function ($S(t) \propto A\cdot e^{-k_{HT} t}$); the ensuing electron injection and triplet formation kinetics are simultaneously fitted using two-exponential decay functions ($S(t) \propto A_1\cdot e^{-k_{ET,1} t} + A_2\cdot e^{-k_{ET,2} t}$). The fitting parameters are listed in Supplementary Table 3, and the rate constants are converted to time constants in the table.

Model for NC-NCA complexes. According to the TA and PL measurements, a kinetic fitting model is proposed as follows: upon selective photoexcitation of $CsPbBr_3$ NCs in NC-NCA complexes, a sub-ensemble of exited-states (NC*-NCA) quickly decay in 200 ps primarily

through ultrafast hole trapping. The rest NC*-NCA without trap states decay via direct transfer of the band edge exciton energy of NCs to the triplet states of NCA, while the TA signal of ^{3}NCA* grows in. Thus, we simultaneously fit the PL and ^{3}NCA* formation kinetics in NC-NCA complexes with a two-exponential decay function ($S(t) \propto A_1 \cdot e^{-k_{PL,1}t} + A_2 \cdot e^{-k_{PL,2}t}$).The fitting parameters are listed in Supplementary Table 4, and the rate constants are converted to time constants in the table.

The rate constants of TET from NC-to-NCA are calculated from the following equation: $\langle k\rangle_{TET} = \langle k\rangle_{NC\text{-}NCA,PL} - \langle k\rangle_{NC,PL}$, where $\langle k\rangle_{NC\text{-}NCA,PL}$ and $\langle k\rangle_{NC,PL}$ are the average rate constants obtained from exponential fitting of $CsPbBr_3$ PL decay with and without, respectively, surface-anchored NCA acceptors. The TET yields (Φ_{TET}) can be calculated using the following equation: $\Phi_{TET} = \langle k\rangle_{TET}/\langle k\rangle_{NC\text{-}NCA,PL}$.

Supplementary Note 3. Estimation of triplet formation yields

The $CsPbBr_3$ NC-to-PAH triplet formation yields were determined by using ultrafast TA data. For the $CsPbBr_3$ control spectra, the ΔA at the wavelength of exciton bleach (XB) and the corresponding ground state molar extinction coefficient (ε_1) for the $CsPbBr_3$ NCs at the XB peak (~615000 $M^{-1}cm^{-1}$)[13] were used to calculate the concentration of NC excited states (NC^*) generated. For the NC-PAH samples, the experiments were performed under identical experimental conditions. The concentration of $^3PAH^*$ formed was determined using the maximum ΔA at ~425 nm for NCA (465 nm for TCA) and its corresponding triplet molar extinction coefficient ε_2.[14] Thus, the comparison of the concentration of $CsPbBr_3$ NC^*

generated by the laser pulse in absence of PAH acceptor and the concentration of $^3PAH^*$ generated from TET permitted the determination of the triplet formation yield:

$$\Phi_{TET} = \frac{[^3PAH^*]}{[NC^*]} = \frac{\Delta A_{^3PAH^*} / \varepsilon_2}{\Delta A_{NC^*} / (\varepsilon_1 / 2)} = \frac{\Delta A_{^3PAH^*} \varepsilon_1}{2\Delta A_{NC^*} \varepsilon_2} \quad (14).$$

Note that the factor of 2 accounts for the two-fold spin-degeneracy of the band edge state in $CsPbBr_3$ NCs, that is, one exciton in a NC only bleaches half of its absorption. The calculated TCA and NCA triplet formation yields are listed as follows.

$$\Phi_{TET(NC-TCA)} = \frac{\Delta A_{^3\mathrm{TCA}^*} \varepsilon_1}{2\Delta A_{NC^*} \varepsilon_2} = \frac{1.25 * 615000}{2 * 13 * 31200} = 94.8\% \quad (15),$$

$$\Phi_{TET(NC-NCA)} = \frac{\Delta A_{^3\mathrm{NCA}^*} \varepsilon_1}{2\Delta A_{NC^*} \varepsilon_2} = \frac{0.4 \times 615000}{2 \times 14.5 \times 13200} = 64.3\% \quad (16).$$

Supplementary Reference

1 Ravi, V. K., Markad, G. B. & Nag, A. Band Edge Energies and Excitonic Transition Probabilities of Colloidal CsPbX3 (X = Cl, Br, I) Perovskite Nanocrystals. *ACS Energy Letters* **1**, 665-671 (2016).

2 Kucur, E., Riegler, J., Urban, G. A. & Nann, T. Determination of quantum confinement in CdSe nanocrystals by cyclic voltammetry. *J. Chem. Phys.* **119**, 2333-2337 (2003).

3 Haram, S. K., Quinn, B. M. & Bard, A. J. Electrochemistry of CdS Nanoparticles: A Correlation between Optical and Electrochemical Band Gaps. *J. Am. Chem. Soc.* **123**, 8860-8861 (2001).

4 Luo, X., Liang, G., Wang, J., Liu, X. & Wu, K. Picosecond multi-hole transfer and microsecond charge-separated states at the perovskite nanocrystal/tetracene interface. *Chem. Sci.* **10**, 2459-2464 (2019).

5 Huang, Z. *et al.* PbS/CdS Core–Shell Quantum Dots Suppress Charge Transfer and Enhance Triplet Transfer. *Angew. Chem. Int. Ed.* **56**, 16583-16587 (2017).

6 Zhu, H. *et al.* Auger-Assisted Electron Transfer from Photoexcited Semiconductor Quantum Dots. *Nano Lett.* **14**, 1263-1269 (2014).

7 Rehm, D. & Weller, A. Kinetics of Fluorescence Quenching by Electron and H-Atom Transfer. *Israel Journal of Chemistry* **8**, 259-271 (1970).

8 Ghosh, I., Shaikh, R. S. & König, B. Sensitization-Initiated Electron Transfer for Photoredox Catalysis. *Angew. Chem. Int. Ed.* **56**, 8544-8549 (2017).

9 Banerjee, T. *et al.* Diphenylisobenzofuran Bound to Nanocrystalline Metal Oxides:

Excimer Formation, Singlet Fission, Electron Injection, and Low Energy Sensitization. *J. Phys. Chem. C* **122**, 28478-28490 (2018).

10 Oxman, J. D. *et al.* Evaluation of initiator systems for controlled and sequentially curable free-radical/cationic hybrid photopolymerizations. *Journal of Polymer Science Part A: Polymer Chemistry* **43**, 1747-1756 (2005).

11 Tvrdy, K., Frantsuzov, P. A. & Kamat, P. V. Photoinduced electron transfer from semiconductor quantum dots to metal oxide nanoparticles. *Proc. Natl. Acad. Sci.* **108**, 29-34 (2011).

12 Protesescu, L. *et al.* Nanocrystals of Cesium Lead Halide Perovskites (CsPbX3, X = Cl, Br, and I): Novel Optoelectronic Materials Showing Bright Emission with Wide Color Gamut. *Nano Lett.* **15**, 3692-3696 (2015).

13 Maes, J. *et al.* Light Absorption Coefficient of CsPbBr3 Perovskite Nanocrystals. *J. Phys. Chem. Lett.* **9**, 3093-3097 (2018).

14 Montalti, M., Credi, A., Prodi, L. & Gandolfi, M. T. *Handbook of photochemistry*. Vol. 3rd ed., CRC, Taylor & Francis, Boca Raton (CRC press, 2006).
